# Efficacy, Safety, and Pharmacokinetics of Golimumab in Children with Moderately-To-Severely Active Ulcerative Colitis: Results from the PURSUIT 2 Study

**DOI:** 10.1093/ibd/izaf322

**Published:** 2026-01-08

**Authors:** Dan Turner, Kathleen G Lomax, Gigi Veereman, Anne M Griffiths, Jaroslaw Kierkuś, Ben Kang, Katherine Berezny, Lakshmi Padgett, Gary Mao, Yevgeny Zitser, Richard S Strauss, Jeroen Verhoeven, Omoniyi J Adedokun, Jeffrey S Hyams

**Affiliations:** The Juliet Keidan Institute of Pediatric Gastroenterology and Nutrition, Shaare Zedek Medical Center, The Hebrew University of Jerusalem, Jerusalem, 91031, Israel; Johnson & Johnson, Spring House, PA, 19477, United States; Universitair Ziekenhuis, Vrije Universiteit Brussel, Brussels, 1090, Belgium; The Hospital for Sick Children, University of Toronto, Toronto, Ontario, ON M5G 1X8, Canada; The Children’s Memorial Health Institute, Warsaw, 04-730, Poland; Kyungpook National University Chilgok Hospital, Daegu, 41944, South Korea; Johnson & Johnson, Spring House, PA, 19477, United States; Johnson & Johnson, Spring House, PA, 19477, United States; Johnson & Johnson, Spring House, PA, 19477, United States; Johnson & Johnson, Spring House, PA, 19477, United States; Kharkiv State University, Kharkiv Oblast, 61022, Ukraine; Johnson & Johnson, Spring House, PA, 19477, United States; Department of Clinical Pharmacology, Johnson & Johnson, Beerse, 2340, Belgium; Johnson & Johnson, Spring House, PA, 19477, United States; Connecticut Children’s Medical Center, Hartford, CT, 06106, United States

**Keywords:** golimumab concentrations, clinical response, clinical remission, endoscopic improvement, pediatric, anti-TNFα, inflammatory bowel disease

## Abstract

**Background:**

Golimumab is an anti-TNFα biologic agent that has been approved for adults with moderately-to-severely active ulcerative colitis (UC). We investigated the efficacy, safety, and pharmacokinetics (PK) of golimumab in biologic-naïve children with moderately-to-severely active UC.

**Methods:**

The prospective, multicenter, open-label PURSUIT 2 study enrolled biologic-naïve children (2 to <18 years) with moderately-to-severely active UC (Mayo score 6-12; endoscopic subscore ≥2) despite conventional treatment. During the 6-week induction phase, patients received subcutaneous (SC) golimumab dosed by weight (<45 kg: 120/60 mg/m^2^; ≥45 kg: 200/100 mg) at weeks 0/2. Week 6 clinical responders (Mayo score decrease from baseline ≥30% and ≥3 points, with either a decrease in the rectal bleeding subscore of ≥1 or a rectal bleeding subscore of 0/1) continued golimumab dose q4w during the 48-week maintenance phase. The primary endpoint was clinical remission (Mayo score ≤2 points with no individual subscore >1) at week 6. Efficacy and PK data are presented alongside a reference adult UC population who received golimumab SC 200/100 mg at weeks 0/2 and 100 mg q4w thereafter.

**Results:**

Of the 69 patients (mean age, 13.4 ± 3.3 years) enrolled, 31.9% (22/69) were in clinical remission at week 6, and of those, 54.5% (12/22) remained in clinical remission at week 54. At week 54, 31.7% (13/41) of week 6 clinical responders achieved clinical remission. Additionally, 33.3% achieved clinical remission by the Pediatric Ulcerative Colitis Activity Index (PUCAI), 56.5% clinical response by Mayo, 40.6% endoscopic improvement, and 7.2% endoscopic remission at week 6. Of those who were clinical responders at week 6, 34.1% achieved clinical remission by PUCAI, 31.7% corticosteroid-free clinical remission by Mayo, 36.6% endoscopic improvement, and 9.8% endoscopic remission at week 54. Serum golimumab concentrations through week 6 were comparable to the reference adult UC population. Through week 54, 40.6% reported serious adverse events and 13.0% serious infections. No new safety concerns were identified.

**Conclusions:**

Overall, the results of the PURSUIT 2 study support golimumab treatment for children with moderately-to-severely active UC.

**ClinicalTrials.gov ID:** NCT03596645

Key Messages

- **What is already known?** 
  Subcutaneous (SC) golimumab is safe and efficacious for adults with moderately-to-severely active ulcerative colitis (UC); however, it is not yet approved for children with UC.
- **What is new here?** 
  The open-label PURSUIT 2 study found SC golimumab induction and maintenance treatment to be efficacious and well tolerated in biologic-naïve children with moderately-to-severely active UC despite conventional treatment, with pharmacokinetics in children comparable to those of a reference adult UC population.
- **How can this study help patient care?** 
  SC golimumab could be a treatment option for children with UC.

## Introduction

Approximately 11% of patients with ulcerative colitis (UC) present before the age of 18 years.^1^ In a multicenter cohort study conducted in Canada, of the 392 children and adolescents who had a new diagnosis of UC or inflammatory bowel disease (IBD)-unclassified, 24% were diagnosed by 10 years old and 6% by 6 years old.^2^ Pediatric and adult populations with UC share similar clinical features, disease course, and treatment response; however, more extensive and severe UC and a greater prevalence of pancolitis have been observed for pediatric-onset UC compared to adult-onset UC.^3–6^

Although there are multiple advanced therapies in several distinct drug classes approved to treat adults with UC, infliximab and adalimumab are currently the only approved pharmacologic treatment options for children with UC.^7–9^ The guidelines for the management of children with UC from the European Crohn’s and Colitis Organization and European Society of Paediatric Gastroenterology, Hepatology, and Nutrition recommend that infliximab be considered for both induction and maintenance for children with chronically active or steroid-dependent UC that is not controlled by 5-aminosalicylate and thiopurines, and that adalimumab be considered for children who are intolerant to or become nonresponsive to infliximab.^10^ Nonetheless, not all children achieve long-term remission with infliximab or adalimumab. For example, in a study of children with active UC treated with infliximab, the remission rate was 28.6% at week 54 with maintenance treatment (every 8 weeks).^7^ In the ENVISION I study of children with moderately-to-severely active UC treated with adalimumab, the remission rate was 29% at week 52 in the standard dose arm.^8^ As such, a need exists for additional safe and efficacious treatments for children with moderately-to-severely active UC.

Golimumab (Simponi^®^, Janssen Biotech, Inc., Horsham, PA) is an antitumor necrosis factor alpha (TNFα) agent administered by the subcutaneous (SC) route.^11^ In the Phase 2/3 “Program of Ulcerative Colitis Research Studies Utilizing an Investigational Treatment” (PURSUIT) double-blind, placebo-controlled study of SC golimumab in adults with moderately-to-severely active UC despite conventional treatment (PURSUIT-SC; NCT00487539), golimumab induction therapy led to clinically meaningful improvements compared with placebo in the signs and symptoms of UC at induction week 6 and was well tolerated.^12^ In the maintenance phase (PURSUIT-M; NCT00488631), continued golimumab therapy was shown to maintain clinical response through week 54 among those who responded to golimumab induction therapy.^13^ On the basis of these studies, SC golimumab was approved for the treatment of adults with moderately-to-severely active UC in both the United States and European Union in 2013.^14^

In children, the Phase 1b, multicenter, open-label, pharmacokinetics (PK) study of SC golimumab recruited 35 biologic-naïve children 6 to 17 years old with moderately-to-severely active UC (PURSUIT PEDS PK; NCT01900574).^15^ The study showed similar golimumab PK and efficacy endpoints as in adult patients. In the extension phase of PURSUIT PEDS PK, continued clinical benefit was observed in children with UC who received SC golimumab maintenance therapy, with clinical remission being achieved by half of the patients at week 110.^16^

The PURSUIT 2 study aimed to further evaluate the efficacy, safety, and PK of SC golimumab induction and maintenance therapy in biologic-naïve children (2 to <18 years old) with moderately-to-severely active UC despite conventional therapy.

## Methods

### Study Design

This prospective, multicenter, open-label study (PURSUIT 2; ClinicalTrials.gov ID: NCT03596645; registration date: July 13, 2018) assessed the efficacy, safety, and PK of SC golimumab in children with moderately-to-severely active UC (Figure S1). The study was conducted from December 2018 to April 2024 at 26 sites in Belgium, Poland, France, Italy, Spain, Korea, the United States, Brazil, and Israel.

In response to a request from health authorities, an infliximab reference arm was included in the original study protocol (May 2018) to assess assay sensitivity compared to historical data from a study of infliximab in children with UC (NCT00336492). Protocol Amendment 4 (March 2021) terminated enrollment into the infliximab arm for the following reasons: interpretable golimumab study data would be available without the infliximab arm, limited ability to interpret the infliximab data, and updated study feasibility assessments. This amendment was discussed with health authorities, and it was accepted that enrollment into the infliximab arm could be stopped. Upon implementation of the amendment, no additional patients were randomized to infliximab, all newly enrolled patients received golimumab, and assay sensitivity was not assessed. Fourteen patients received infliximab treatment before Protocol Amendment 4 was implemented; no formal comparisons between golimumab and infliximab were planned or performed and are, therefore, not included in this report.

During the 6-week induction phase (week 0 to week 6), patients ≥30 kg received body weight-based golimumab SC, or infliximab intravenous (IV), in a 3:1 ratio (by permuted block randomization), at weeks 0 and 2 (before Protocol Amendment 4) or golimumab SC only (after Amendment 4). Patients <30 kg were not randomized and received only weight-based golimumab. Golimumab SC dosing (weight- or body surface area [BSA]-based doses) was as follows: 200 mg at week 0 and 100 mg at week 2 (if weight ≥45 kg); 120 mg/m^2^, up to a maximum of 200 mg at week 0, and 60 mg/m^2^, up to a maximum of 100 mg at week 2 (if weight <45 kg) (Figure S1). During the 48-week maintenance phase (week 6 [post-dose] to week 54), patients in clinical response to golimumab (see definition below) at week 6 continued to receive SC golimumab 100 mg or 60 mg/m^2^ every 4 weeks (q4w) through week 50. Patients not in clinical response at week 6 could (at the discretion of the investigator) receive 2 additional doses of SC golimumab (i.e., at week 6 and week 10), and those who were partial Mayo responders at week 14 (defined as a decrease from the week 0 partial Mayo score of ≥3 points) continued to receive SC golimumab 100 mg or 60 mg/m^2^ q4w through week 50. Patients who were not in partial Mayo response at week 14 were withdrawn from the study. The only dose adjustment permitted was to maintain the dose to match the body weight- or BSA-based dosing; otherwise, no dose adjustment was allowed in this study through week 54.

### Historical Reference Adult UC Population

No placebo arm was included in this study because it is unethical in children with IBD.^17^ Thus, we present the efficacy and PK results of this study alongside a historical reference adult UC population from the Phase 2/3 placebo-controlled golimumab studies in adults with moderately-to-severely active UC (PURSUIT-SC and PURSUIT-M).^12^^,^^13^ This approach was used in a published golimumab study in children with moderately-to-severely active UC (PURSUIT PEDS PK), the precursor study to PURSUIT 2.^15^ PURSUIT-SC consisted of a double-blind, randomized, placebo-controlled Phase 2 dose-finding study and a Phase 3 confirmation study. Adults with moderately-to-severely active UC (*N* = 1064) were given placebo or SC golimumab (weeks 0/2: 100 mg/50 mg [Phase 2 only], 200 mg/100 mg, or 400 mg/200 mg).^12^ Data are shown for the placebo and golimumab SC 200/100 mg groups only; some of these results have not been previously published. PURSUIT-M was a Phase 3, 54-week, double-blind, randomized, placebo-controlled maintenance study in which 464 adults who completed either of 2 golimumab induction studies (PURSUIT-SC or PURSUIT-IV [NCT00488774])^12^^,^^18^  and were golimumab induction therapy responders were given placebo or SC golimumab (50 mg or 100 mg q4w) through week 52.^13^ Data are shown for the placebo and golimumab SC 100 mg groups only; some of these results have not been previously published. No comparative statistical analyses were performed between children and adult UC populations.

### Eligibility

Eligible patients were 2 to <18 years old (weight ≥10 kg and height >70 cm) with a confirmed diagnosis of UC (≥1 month before screening). Patients had moderately-to-severely active UC, defined as a baseline Mayo score of 6 to 12 (inclusive) and endoscopy subscore ≥2 assessed by the local endoscopist. Patients also had to meet ≥1 of the following medication criteria: (1) were receiving current treatment with oral corticosteroids or immunomodulators (i.e., 6-mercaptopurine, methotrexate, or azathioprine); (2) had a history of failure to respond to or tolerate, or have a medical contraindication to oral or IV corticosteroids or the immunomodulators; (3) were unable to reduce corticosteroids without UC symptoms returning; or (4) in the past year, needed ≥3 courses of corticosteroids. In addition, patients must not have ever received any anti-TNFα biologic agent. Patients with very severe active UC disease and/or were currently hospitalized for UC disease exacerbation at study screening and who had a Mayo score of 12 were not eligible for inclusion. Also excluded were patients with a history of insufficient liver or renal function or any other significant medical condition.

### Clinical Outcomes

#### Evaluations

Four subscores comprise the Mayo score (stool frequency, rectal bleeding, endoscopy findings, and physician’s global assessment), each rated 0 (normal) to 3 (severe), with a total Mayo score of 0 to 12 (scores 3-5 = mildly active UC; 6-10 = moderately active UC; 11-12 = severely active UC) and a partial Mayo score (without the endoscopy subscore) of 0 to 9.^19^

Six scales comprise the Pediatric Ulcerative Colitis Activity Index (PUCAI), a noninvasive measure of UC disease activity (abdominal pain, rectal bleeding, stool consistency, number of stools, nocturnal bowel movement, and activity level), with a total score of 0 to 85 (score <10 = remission; decrease of 20 points = minimally clinically important change).^20^

#### Outcome Assessments

The primary efficacy outcome was clinical remission (Mayo), defined as a Mayo score of ≤2 points with no individual subscore >1, at week 6.^21^ Other efficacy outcomes at week 6 included the following: clinical remission, defined as PUCAI <10; clinical response, defined as a Mayo score decrease from baseline of ≥30% and ≥3 points, with either a decrease from baseline in the rectal bleeding subscore of ≥1 or a rectal bleeding subscore of 0 or 1; endoscopic improvement (or endoscopic healing, per protocol), defined as a Mayo endoscopy subscore of 0 or 1; and endoscopic remission (or endoscopic normalization, per protocol), defined as a Mayo endoscopy subscore of 0.

Maintenance phase efficacy outcomes were collected for patients who were in clinical response at week 6 (see definition above) and received ≥1 dose of golimumab. Key efficacy outcomes at week 54 included the following: clinical remission (Mayo), clinical remission (PUCAI), endoscopic improvement, and endoscopic remission (see definitions above); corticosteroid-free clinical remission among week 6 clinical responders, defined as corticosteroid-free clinical remission (Mayo) at week 54 among patients who were not receiving corticosteroids for at least 12 weeks before week 54 (includes oral, parenteral, and rectal routes). Maintenance of clinical remission (Mayo), defined as clinical remission (Mayo) at week 54 among patients who were in clinical remission (Mayo) at week 6, was also assessed.

### Concomitant Medications

The following concomitant mediations for UC were allowed: 5-aminosalicylates, corticosteroids (including budesonide), and immunomodulators (i.e., 6-mercaptopurine, azathioprine, or methotrexate). Dosages of these medications must have been stable for 2 weeks before the first administration of golimumab at week 0. For patients receiving 5-aminosalicylates, the dose must have remained stable through week 54 (except for weight-based adjustments, as treatment of a documented UC flare after week 6, or dose reduction or cessation because of toxicity or medical necessity). Immunomodulators (i.e., 6-mercaptopurine, azathioprine, or methotrexate) could not have been started between week 0 and week 6. For patients receiving immunomodulators, the dose must not have been increased (except for weight-based adjustments) through week 6. Immunomodulators could have been discontinued at any time (preferred that they be continued at least through week 14), and corticosteroids (including budesonide) could have been tapered beginning at week 0 (preferred to delay the stopping of corticosteroids until after the week 6 evaluation). Corticosteroids could not have been started to treat UC disease flare until after week 6 study assessments.

### Safety

Adverse events (AEs; at the visit and those occurring between evaluation visits) were reported for the induction phase (week 0 to week 6), the maintenance phase (week 6 [post-dose] to week 54), and the entire study period (week 0 through week 54). An AE was defined as any untoward medical event in a patient receiving golimumab, whether or not it was caused by the treatment. A serious AE was defined as any untoward medical event (at any dose of study medication) that led to death, was life threatening, required or prolonged hospitalization, caused lasting or significant disability or incapacity, was a congenital anomaly or birth defect, was a suspected transmission of any infectious agent through the study medication, or was otherwise considered medically important. Infections and serious infections were deemed as such by the investigator.

### PK

Venous blood samples were collected for measurement of serum golimumab concentrations over 54 weeks. A validated streptavidin-coated plate-based “sandwich” electrochemiluminescence immunoassay on the Meso Scale Discovery platform (Meso Scale Discovery, Gaithersburg, MD) was used to determine serum golimumab concentrations. The lowest quantifiable concentration was 0.03905 µg/mL.

### Statistical Methods

Sample size estimation was included in the study protocol, which was finalized before the first patient was enrolled. Sample size for the study was determined to have a sufficient number of patients assigned to the golimumab treatment arm to demonstrate with sufficient power that golimumab is an effective therapy in children with UC relative to a historical placebo control, as assessed by clinical remission at week 6 (by the Mayo score). The historical placebo rate was estimated to be 8.3% (95% CI, 6.6% to 10.0%), based on a meta-analysis of seven similarly designed phase 3 studies in adults with moderately-to-severely active UC, including two studies of golimumab, two studies of infliximab, two studies of adalimumab, and one study of vedolizumab (Table S1). The upper confidence interval (CI) limit represents an estimate of the upper limit of placebo response. If the lower CI limit of the active treatment arm is above the upper CI limit of placebo response, one could reasonably conclude that the active treatment is more effective than placebo. With 60 golimumab-treated patients, the probability of meeting the above success criterion would be greater than 80%, assuming the remission rate at week 6 for golimumab-treated patients was at least 22.5%. Additional details of the historical placebo control meta-analysis and sample size determination are provided in the Supplemental Materials. In addition, the Fisher Information Matrix-based optimal design analysis indicated that PK data from 60 golimumab-treated patients would be sufficient to adequately characterize the PK of golimumab in children with UC.

Data were summarized with descriptive statistics: mean (standard deviation) and median (range) for continuous variables, and counts (percentages) for categorical variables.

Treatment failure rules for all efficacy endpoints included the following: patients who had a disallowed change in UC medication, UC surgery (ostomy or colectomy), used a rescue medication after a UC flare (after week 6), or stopped taking the study medication due to lack of efficacy or an AE of worsening of UC before the week 6/week 54 visit were considered not to have achieved the endpoint. If a patients stopped taking study medication because of COVID-19-related reasons, data were used as available. For all analyses, patients with missing data for binary endpoints were considered to not have achieved their respective endpoint, as follows: patients who were missing all 4 Mayo subscores (clinical remission [Mayo] or clinical response), >3 PUCAI subscores (clinical remission [PUCAI]), all 4 Mayo subscores or endoscopy score (corticosteroid-free clinical remission [Mayo]), or endoscopy score (endoscopic improvement, endoscopic remission) at week 6/week 54 were considered not to have achieved the endpoint at week 6/week 54. For corticosteroid-free clinical remission (Mayo) at week 54, if a corticosteroid use value was missing, the last value was carried forward. The CIs use the asymptotic formula based on the normal approximation to the binomial distribution.

Safety was assessed by summarizing the frequencies and types of AEs.

Descriptive statistics (median) of the serum golimumab concentrations were calculated at each sampling time point and summarized over time. This analysis included patients who received ≥1 golimumab dose and had ≥1 valid blood sample drawn for PK analysis during the induction phase.

## Results

### Baseline Patient Characteristics, Medication History, and Patient Disposition

**Figure 1.**
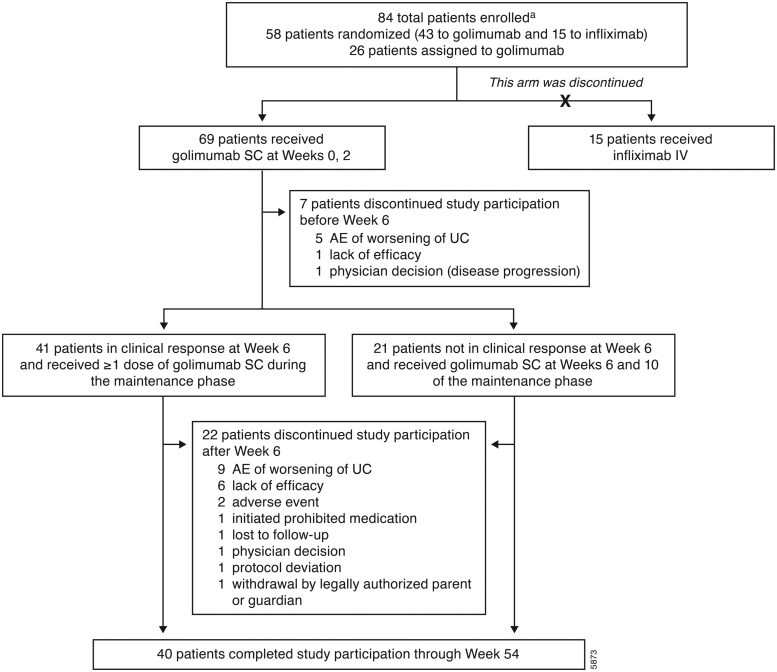
PURSUIT 2 patient disposition through week 54. ^a^Key inclusion criteria: 2 to <18 years old (weight ≥10 kg and height >70 cm); confirmed diagnosis of UC for ≥1 month; moderately-to-severely active UC, defined as a baseline Mayo score of 6 to 12 and endoscopy subscore ≥2, despite conventional therapy; and biologic-naïve. Abbreviations: AE, adverse event; IV, intravenous; SC, subcutaneous; UC, ulcerative colitis.

Of the 84 total patients enrolled, 69 received golimumab **­(**Figure 1**)**. Thirty-two (46.4%) of the patients were from Europe (Belgium [*n* = 3], Poland [*n* = 16], France [*n* = 1], Italy [*n* = 9], Spain [*n* = 3]), with the remainder from Korea (*n* = 12), the United States (*n* = 4), Brazil (*n* = 11), and Israel (*n* = 10). Most patients were >12 years old (78.3%; mean age, 13.4 ± 3.3 years), with only two patients being 2 to 5 years old **(**Table 1**)**. Seventeen (24.6%) patients weighed <45 kg (8 of whom were <30 kg) and received golimumab BSA-based dosing. Fifty-two (75.4%) patients weighed ≥45 kg and received the golimumab adult fixed dose regimen. The median (range) duration of disease was 1.43 (0.2-7.8) years. On the basis of Mayo score, most patients (91.3%) had moderate UC disease activity. Two patients were inadvertently enrolled with Mayo score of 5 due to miscalculation, and thus, were categorized with mild UC. Both patients had endoscopy subscores ≥2 (1 subscore of 2 [moderate UC activity] and 1 subscore of 3 [severe UC activity]) and were allowed to remain in the study. On the basis of PUCAI score, 69.5% of patients had moderately-to-severely active UC. One patient had no disease based on PUCAI score of <10. At baseline, a majority of patients (97.1%) were receiving UC-related medications: corticosteroids (52.2%), immunomodulators (47.8%), or 5-aminosalicylate (88.4%).

**Table 1.** Baseline characteristics and medication history at induction baseline.

	Golimumab SC *N* = 69
**Age in years, mean (SD)**	13.4 (3.3)
**Age category in years**	
**2 to <6**	2 (2.9)
**6 to <12**	13 (18.8)
**12 to <18**	54 (78.3)
**Female**	37 (53.6)
**Race**	
**White**	50 (72.5)
**Asian**	12 (17.4)
**Black or African American**	2 (2.9)
**Other**	5 (7.2)
**Weight category in kg**	
**<45**	17 (24.6)
**<30 kg**	8 (11.6)
**≥30 kg to <45**	9 (13.0)
**≥45 kg**	52 (75.4)
**UC duration in years, median (range)**	1.43 (0.2-7.8)
**Extent of UC^a^**	
**Left-sided**	32 (46.4)
**Extensive**	37 (53.6)
**Mayo score, median (range)**	7.0 (5-11)
**Mayo score ≥3 to ≤5 (mild)**	2 (2.9)^b^
**Mayo score ≥6 to ≤10 (moderate)**	63 (91.3)
**Mayo score >10 (severe)**	4 (5.8)
**PUCAI score, median (range)**	40.0 (0-85)
**PUCAI score <10 (no disease)**	1 (1.4)
**PUCAI score ≥10 to ≤34 (mild)**	20 (29.0)
**PUCAI score >34 to <65 (moderate)**	37 (53.6)
**PUCAI score ≥65 (severe)**	11 (15.9)
**C-reactive protein, mg/L, median (range)**	1.59 (0.1-92.4)^c^
**Fecal calprotectin, mg/kg, median (range)**	1590.0 (36-36 000)^d^
**UC medication at induction baseline^e^**	67 (97.1)
**Corticosteroids**	36 (52.2)
**6-mercaptopurine or azathioprine**	33 (47.8)
**Oral 5-aminosalicylate**	61 (88.4)

Values are n (%) unless otherwise specified.

a Based on local endoscopy.

b Two patients were inadvertently enrolled with Mayo score of 5 due to miscalculation, and thus, were categorized with mild UC. Both patients had endoscopy subscores ≥2 (1 subscore of 2, 1 subscore of 3) and were allowed to remain in the study.

c *N* = 68.

d *N* = 63.

e Patients could appear in more than 1 category.

Abbreviations: PUCAI, Pediatric Ulcerative Colitis Activity Index; SC, subcutaneous; SD, standard deviation; UC, ulcerative colitis.

Seven of 69 (10.1%) and 22 of 62 (35.5%) patients discontinued study participation during the induction phase and maintenance phase, respectively **(**Figure 1**)**. The most common reason for study discontinuation was an AE of worsening of UC disease during the induction phase (5 patients, 7.2%) and maintenance phase (9 patients, 14.5%).

### Clinical and Endoscopic Outcomes

**Figure 2.**
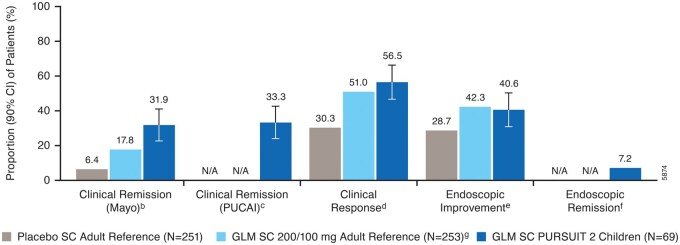
Clinical and endoscopic outcomes at week 6.^a^ ^a^Patients who had a prohibited change in UC medication, an ostomy or colectomy, or discontinued study agent due to lack of efficacy or an AE of worsening of UC before the week 6 visit were considered not to have achieved the endpoint; data after a discontinuation of study agent due to COVID-19-related reasons were used as available. ^b^Clinical remission (Mayo) was defined as a Mayo score ≤2 points, with no individual subscore >1; patients who were missing all 4 Mayo subscores at week 6 were considered not to have achieved the endpoint. ^c^Clinical remission (PUCAI) was defined as a PUCAI score <10; patients who were missing >3 PUCAI subscores at week 6 were considered not to have achieved the endpoint. ^d^Clinical response was defined as a Mayo score decrease from baseline of ≥30% and ≥3 points, with either a decrease from baseline in the rectal bleeding subscore of ≥1 or a rectal bleeding subscore of 0 or 1; patients who were missing all 4 Mayo subscores at week 6 were considered not to have achieved the endpoint. ^e^Endoscopic improvement was defined as a Mayo endoscopy subscore of 0 or 1; patients who were missing endoscopy score at week 6 were considered not to have achieved the endpoint. ^f^Endoscopic remission was defined as a Mayo endoscopy subscore of 0; patients who were missing endoscopy score at week 6 were considered not to have achieved the endpoint; note: 90% CI was not calculated for this outcome at week 6. ^g^In the adult reference study, patients received golimumab SC 200 mg at week 0 and 100 mg at week 2.^12^ Abbreviations: AE, adverse event; CI, confidence interval; GLM, golimumab; N/A, not available; PUCAI, Pediatric Ulcerative Colitis Activity Index; SC, subcutaneous; UC, ulcerative colitis.

At week 6, 31.9% (22 of 69) of the golimumab-treated children were in clinical remission by Mayo (Figure 2). The lower limit of the 90% CI (22.5%-41.1%) of the remission rate was >10%, suggesting superior golimumab treatment effect in children over the historical placebo effect in adults. In the adult reference population, the clinical remission rates at week 6 were 17.8% (45 of 253) in the golimumab 200/100 mg group and 6.4% (16 of 251) in the historical placebo group. Additionally, of the golimumab-treated children, 33.3% achieved clinical remission by PUCAI, 56.5% clinical response by Mayo, 40.6% endoscopic improvement, and 7.2% endoscopic remission at week 6, which were comparable to the adult reference golimumab population for the available endpoints.

**Figure 3.**
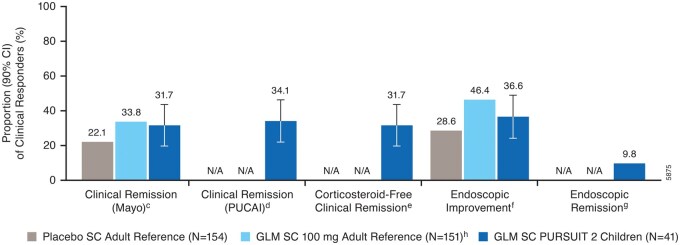
Clinical and endoscopic outcomes at week 54 among patients in clinical response at week 6.^a, b^ ^a^Included patients who were in clinical response (defined as a Mayo score decrease from baseline of ≥30% and ≥3 points, with either a decrease from baseline in the rectal bleeding subscore of ≥1 or a rectal bleeding subscore of 0 or 1) at week 6 and who received ≥1 dose of golimumab during the maintenance phase. ^b^Patients who had a prohibited change in UC medication, an ostomy or colectomy, used a rescue medication after clinical flare, or discontinued study agent due to lack of efficacy or an AE of worsening of UC before the week 54 visit were considered not to have achieved the endpoint; data after a discontinuation of study agent due to COVID-19-related reasons were used as available. ^c^Clinical remission (Mayo) was defined as a Mayo score ≤2 points, with no individual subscore >1; patients who were missing all 4 Mayo subscores at week 54 were considered not to have achieved the endpoint. ^d^Clinical remission (PUCAI) was defined as a PUCAI score <10; patients who were missing >3 PUCAI subscores at week 54 were considered not to have achieved the endpoint. ^e^Corticosteroid-free clinical remission (Mayo) at week 54 among patients who were not receiving corticosteroids for at least 12 weeks before week 54; patients who were missing all 4 Mayo subscores at week 54 were considered not to have achieved the endpoint; patients who had a missing value in corticosteroid use had their last value carried forward. ^f^Endoscopic improvement was defined as a Mayo endoscopy subscore of 0 or 1; patients who were missing endoscopy score at week 54 were considered not to have achieved the endpoint. ^g^Endoscopic remission was defined as a Mayo endoscopy subscore of 0; patients who were missing endoscopy score at week 54 were considered not to have achieved the endpoint; note: 90% CI was not calculated for this outcome at week 54. ^h^In the adult reference study, patients who responded to golimumab induction therapy received golimumab SC 100 mg every 4 weeks from week 2.^13^ Abbreviations: AE, adverse event; CI, confidence interval; GLM, golimumab; N/A, not available; PUCAI, Pediatric Ulcerative Colitis Activity Index; SC, subcutaneous; UC, ulcerative colitis.

At week 54, of the week 6 clinical responders, 31.7% (13 of 41) of the golimumab-treated children achieved clinical remission by Mayo **(**Figure 3**)**. In the adult reference population, the clinical remission rates at week 54 were 33.8% (51 of 151) in the golimumab 200/100 mg group and 22.1% (34 of 154) in the historical placebo group. Additionally, of the golimumab-treated children who were clinical responders at week 6, 34.1% achieved clinical remission by PUCAI, 31.7% corticosteroid-free (not receiving corticosteroids for at least 12 weeks before week 54) clinical remission by Mayo, 36.6% endoscopic improvement, and 9.8% endoscopic remission at week 54. Twelve (17.4%) golimumab-treated patients who were not in clinical response at week 6 received 2 additional doses of golimumab (at week 6 and week 10) and achieved clinical response at week 14 by partial Mayo score (delayed responders). Clinical benefit of continuous maintenance golimumab treatment was observed for the week 14 partial Mayo responders (data not shown). These partial responders were able to maintain clinical response through week 54 and achieve other efficacy outcomes at week 54.

**Figure 4.**
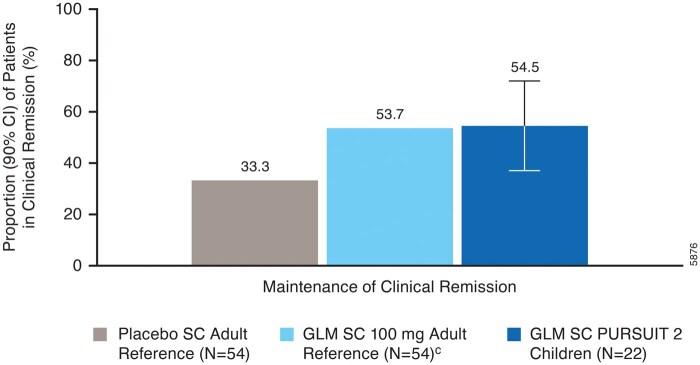
Maintenance of clinical remission at week 54 among patients in clinical remission at week 6.^a, b^ ^a^Patients who had a prohibited change in UC medication, an ostomy or colectomy, used a rescue medication after clinical flare (after week 6), or discontinued study agent due to lack of efficacy or an AE of worsening of UC before the week 6/week 54 visit were considered not to have achieved the endpoint; data after a discontinuation of study agent due to COVID-19-related reasons were used as available. ^b^Clinical remission (Mayo) was defined as a Mayo score ≤2 points, with no individual subscore >1; patients who had all 4 Mayo subscores missing at week 6 (or week 30 [adult reference study]/54) or had a missing endoscopy score at week 30 [adult reference study]/54 were considered not to be in clinical remission at week 30 [adult reference study]/54. ^c^In the adult reference study, patients received golimumab SC 200 mg at week 0, 100 mg at week 2, and 100 mg every 4 weeks thereafter; clinical remission was analyzed at week 30.^12^^,^^13^ Abbreviations: AE, adverse event; CI, confidence interval; GLM, golimumab; SC, subcutaneous; UC, ulcerative colitis.

Of the 22 children who were in clinical remission by Mayo at week 6, 54.5% (12 of 22) remained in clinical remission at week 54 (Figure 4). In the adult reference population, 53.7% (29 of 54) of golimumab-treated patients who were in remission at week 6 maintained clinical remission at week 30 (note: clinical remission at week 54 was not analyzed).

### Safety

A summary of key safety outcomes is provided in Table 2. Through week 54, treatment-emergent AEs were reported among 97.1% of patients, with an average duration of follow-up of 43.4 weeks and an average exposure of 10.1 golimumab administrations. Treatment-emergent AEs were reported among 68.1% of patients during the induction phase (average duration of follow-up, 6.3 weeks; average exposure, 2 administrations) and 93.5% of patients in the maintenance phase (average duration of follow-up, 40 weeks; average exposure, 9 administrations). Through week 54, serious AEs (SAEs) were reported for 40.6% of patients (14.5% of patients in the induction phase; 33.9% of patients in the maintenance phase), with UC (worsening/exacerbation) the most commonly reported SAE. Of the 15 patients (21.7%) who discontinued golimumab due to an AE, 13 discontinued due to UC (worsening/exacerbation). Serious infections were reported for 9 patients (2 cases each of cytomegalovirus colitis and pneumonia, and 1 case each of *Clostridium difficile* infection, COVID-19, pseudomembranous colitis, stump appendicitis, and fungal test positive [Candida]). No malignancies or deaths were reported. Through week 54, the most frequently reported AE was UC (worsening/exacerbation) (Table S2). Of the 42 patients with AEs of UC (worsening/exacerbation), only 1 event was assessed as being “reasonably related” to study drug.

**Table 2.** Key safety outcomes.

	Induction phase (week 0 to week 6) *N* = 69	Maintenance phase (week 6 [Post-Dose] to week 54) *N* = 62^a^	Week 0 through week 54 *N* = 69
**SAEs**	10 (14.5)	21 (33.9)	28 (40.6)
**AEs leading to discontinuation of golimumab**	6 (8.7)	9 (14.5)	15 (21.7)^b^
**AEs**	47 (68.1)	58 (93.5)	67 (97.1)
**Infections**	17 (24.6)	38 (61.3)	43 (62.3)
**Serious infections**	1 (1.4)	9 (14.5)	9 (13.0)^c^
**Injection-site reactions**	2 (2.9)	3 (4.8)	4 (5.8)
**Neoplasms (malignant)**	0	0	0
**Deaths**	0	0	0

Values are *n* (%), where n is the number of patients with ≥1 treatment-emergent event; patients were counted only once for any given event, regardless of the number of times they actually experienced the event. AEs were coded using MedDRA Version 26.1.

a Included patients who received ≥1 dose (complete or partial) of golimumab during the maintenance phase.

b Thirteen patients discontinued due to UC, one due to cytomegalovirus colitis, and one due to fungal test positive (Candida).

c Two cases each of cytomegalovirus colitis and pneumonia, and 1 case each of *Clostridium difficile* infection, COVID-19, pseudomembranous colitis, stump appendicitis, and fungal test positive (Candida) (note: 1 case of “UC worsening” was classified as “infection” though the investigator later confirmed no evidence of infection was found).

Abbreviations: AE, adverse event; MedDRA, Medical Dictionary for Regulatory Activities; SAE, serious adverse event; UC, ulcerative colitis.

### PK

**Figure 5.**
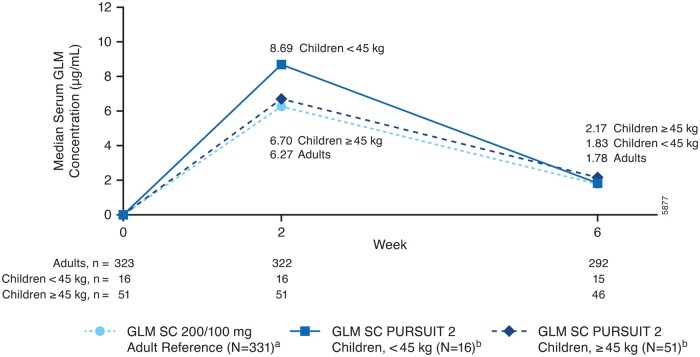
Median serum golimumab concentrations. ^a^Included all randomized patients in the adult reference study (PURSUIT-SC) who received ≥1 golimumab dose at 200/100 mg dose level before week 6 and had ≥1 valid blood sample drawn for PK analysis before week 6 (data on file).^12^ ^b^Included children who received ≥1 SC golimumab dose and had ≥1 valid blood sample drawn for PK analysis during the induction phase (note: 2 patients were excluded because they received an incorrect golimumab SC dose). Abbreviations: GLM, golimumab; PK, pharmacokinetics; SC, subcutaneous.

Median serum golimumab concentrations observed in the pediatric UC population through week 6 were comparable to those observed in the adult reference UC population (Figure 5). Median serum golimumab concentrations through week 6 were generally comparable between patients with body weight <45 kg and those at ≥45 kg.

Median serum golimumab concentrations before administration were consistent from week 10 through week 54, ranging from 1.16 to 1.56 µg/mL. More patients in the highest golimumab concentration quartile (≥2.78 µg/mL) achieved efficacy endpoints at week 6 than those in the lower concentration quartile subgroups (Table S3). No consistent trend was observed in the proportions of patients who achieved efficacy endpoints at week 6 across the 3 lower quartile subgroups (<0.93 µg/mL, ≥0.93 to <1.56 µg/mL, and ≥1.56 to 2.78 µg/mL).

## Discussion

Acknowledging the need for additional efficacious and safe treatment options for children with UC, the PURSUIT 2 study was conducted to evaluate the efficacy, safety, and PK of SC golimumab in biologic-naïve children with moderately-to-severely active UC despite conventional therapy. We presented key results of the PURSUIT 2 study alongside historical data from 2 similarly designed placebo-controlled golimumab induction and maintenance studies in adults with moderately-to-severely active UC,^12^^,^^13^ for whom SC golimumab has been approved in both the United States and European Union. Clinical and endoscopic outcomes during golimumab induction and maintenance treatment, as well as serum golimumab concentrations over 6 weeks in children with UC in the current study were comparable to those observed in the adult UC studies, with no new safety concerns.^12^^,^^13^

The primary endpoint, clinical remission at induction week 6 as assessed by the Mayo score, was achieved by 31.9% of golimumab-treated children, which was higher than the proportion of patients who achieved clinical remission (17.8%) using the same criteria in the adult PURSUIT-SC induction study.^12^ Importantly, 54.5% of the patients who achieved clinical remission at week 6 maintained clinical remission at week 54. The efficacy of SC golimumab therapy in children with UC was supported by the proportions of patients achieving additional clinically meaningful efficacy outcomes at induction week 6 (33.3%-56.5%) and at maintenance week 54 (31.7%-36.6%), including clinical remission using the PUCAI (33.3% at week 6 and 34.1% at week 54), a validated pediatric-specific rating scale.^21^ Of note, 12 (17.4%) golimumab-treated children were considered delayed responders at week 14, as they were not clinical responders at week 6 but became partial Mayo responders at week 14 after receiving additional doses of golimumab at week 6 and week 10. In addition, the therapeutic effects of SC golimumab were observed by the first efficacy assessment at week 6 but may have started earlier during induction therapy since the first efficacy assessment was at week 6 following 2 SC doses at week 0 and week 2. This possibility is supported by results from the PURSUIT-SC adult study in which serum golimumab concentrations peaked at week 2 and golimumab showed therapeutic effects (decreased partial Mayo score and C-reactive protein levels) starting at week 2.^12^

Overall, the safety profile in children through week 54 was consistent with the known safety profile of golimumab in adults with UC.^12^^,^^13^ Additionally, the pattern of AEs reported during the maintenance phase was similar to that of the induction phase. No deaths or malignancies were reported. The most frequently reported treatment-emergent AE in both phases was UC (exacerbation/worsening). It should be noted that in this study, UC flares that led to a patient stopping study medication were considered treatment failures. Twenty-one of 69 children (30.4%) discontinued the study due to an AE of worsening of UC or lack of efficacy. In the golimumab PK study (PURSUIT PEDS PK), 12 of 35 golimumab-treated children (34.3%) discontinued due to lack of efficacy, 11 per protocol due to not achieving clinical response at week 6, and 1 due to UC flare before week 14.^15^

Although no new safety signals were identified in this study, a higher frequency of SAEs through week 54 was observed in this study (33.9%) compared to the corresponding adult PURSUIT-M maintenance study (12.5%),^13^ which may be in part related to allowing nonresponders into the maintenance phase without dose escalation, or because week 6 nonresponders had a higher SAE rate in maintenance than week 6 responders due to a higher occurrence of disease progression under study, or both. Evidence for higher disease hospitalization rates in children with IBD compared with adults may possibly be related to lower thresholds for hospitalization in younger patients.^22–25^

Serum golimumab concentrations through week 6 observed in the overall pediatric UC population were comparable to those observed in the reference adult UC population who received a 200 mg dose at week 0 followed by a 100 mg dose at week 2 and q4w thereafter.^12^ In addition, comparable serum golimumab concentrations were observed in the children weighing less than 45 kg who received the BSA-based dosing and the children weighing 45 kg or more who received the adult fixed dosing, which is different from that observed when mg/kg dosing has been used for all (e.g., infliximab dosing).^26^ Median serum golimumab concentrations before administration were consistent from week 10 through week 54, suggesting that steady-state was achieved approximately 10 weeks after starting golimumab treatment. Steady-state was achieved at approximately 8 weeks after receiving golimumab maintenance doses in the PURSUIT-M adult UC reference study.^13^ Additionally, at week 6, apparent exposure-response trends were observed between serum golimumab concentrations and efficacy outcomes. The proportion of patients who achieved efficacy outcomes tended to be greatest in the highest serum golimumab concentration quartile subgroup during induction and maintenance. In adult patients, positive exposure-response trends were also noted for efficacy outcomes.^12^^,^^13^

Despite the limitations of the open-label study design and lack of parallel placebo- or active-control groups, a strength of PURSUIT 2 was that it was designed to facilitate a comparison of the findings from this study with those of the adult UC golimumab studies.^12^^,^^13^ The dose regimens evaluated in this study were selected to deliver golimumab such that exposure in children would be comparable to that observed in the reference adult UC population. They also had similar study designs and endpoints (i.e., Mayo score remission, used local endoscopic reading, participants were TNFα-naïve and had moderately-to-severely active UC). Nonetheless, as a clinical trial, the findings of PURSUIT 2 may not be generalizable to children with UC evaluated in routine clinical care, especially considering that 72.5% of the patients were white and 46.4% were from European countries. In addition, the study was constrained by having relatively small sample sizes.

In conclusion, SC golimumab has been approved for both induction and maintenance therapy in adults with moderately-to-severely active UC. It has the potential to offer children with UC an efficacious and safe treatment option, given as an SC injection q4w following induction. The PURSUIT 2 study demonstrated that SC golimumab treatment is well tolerated and is efficacious for inducing clinical remission, as well as maintaining it, among biologic-naïve children with moderately-to-severely active UC despite conventional treatment. The efficacy, safety, and PK profiles of golimumab in children with UC were consistent with the known profiles in adults with UC. Overall, the results of the PURSUIT 2 study support SC golimumab induction and maintenance treatment for children with moderately-to-severely active UC.

## Supplementary Material

izaf322_Supplementary_Data

## Data Availability

The data sharing policy of Johnson & Johnson is available at https://innovativemedicine.jnj.com/our-innovation/clinical-trials/transparency. As noted on this site, requests for access to the study data can be submitted through Yale Open Data Access [YODA] Project site at http://yoda.yale.edu.

## Conference Presentation

Part of this work has been previously presented at Digestive Disease week 2025 (May 3-6, 2025; San Diego, CA, USA) and the 57th European Society for Paediatric Gastroenterology, Hepatology and Nutrition (ESPGHAN) Annual Meeting (May 14-17, 2025; Helsinki, Finland).

## Summary

PURSUIT 2 evaluated subcutaneous golimumab in biologic-naïve children with moderately-to-severely active ulcerative colitis (UC). At week 6, 31.9% (22/69) achieved clinical remission, which was maintained in 54.5% (12/22) at week 54. Pharmacokinetics and safety were comparable to adult UC.
