# Supplementary material for: Efficacy, Safety, and Pharmacokinetics of Golimumab in Children with Moderately-To-Severely Active Ulcerative Colitis: Results from the PURSUIT 2 Study
Source: Inflamm Bowel Dis. 2026 Jan 8;32(6):1052–61. doi: 10.1093/ibd/izaf322 (PMC13234503; doi:10.1093/ibd/izaf322)
Supplement: izaf322_Supplementary_Data [file izaf322_supplementary_data.docx]

**SUPPLEMENTAL MATERIALS**

Turner D, et al. Efficacy, Safety, and Pharmacokinetics of Golimumab in Children With Moderately-to-Severely Active Ulcerative Colitis: Results From the PURSUIT 2 Study

**Figure S1. Overview of the PURSUIT 2 Study.**


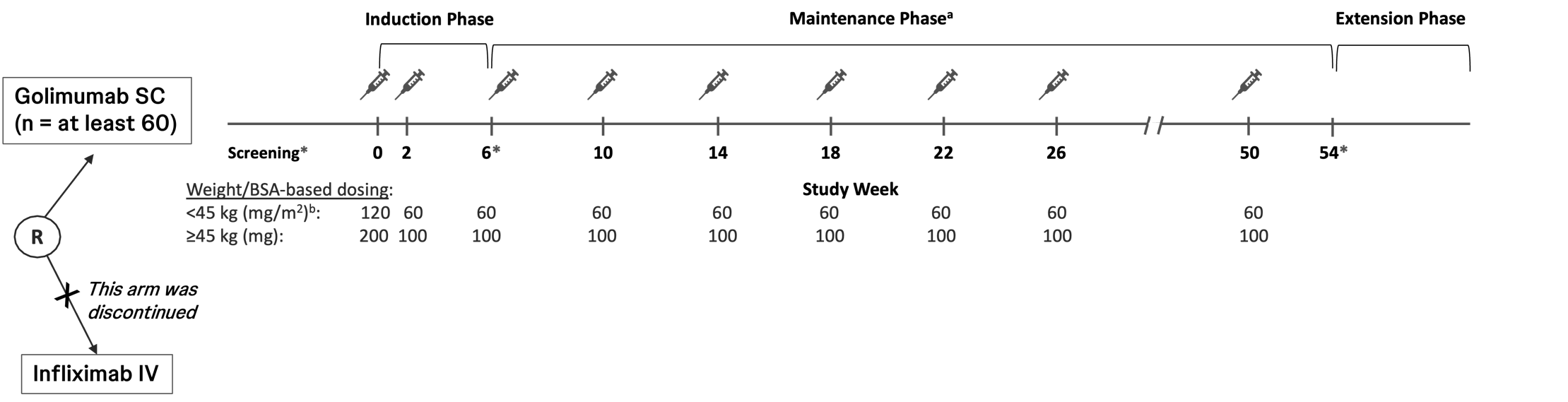


^a^Patients in clinical response to golimumab SC at Week 6 or partial Mayo response at Week 14.

^b^BSA doses were capped at 200 mg for the 120 mg/m^2^ dose and 100 mg for the 60 mg/m^2^ dose.

* Endoscopy

Abbreviations: BSA, body surface area; IV, intravenous; SC, subcutaneous.

**Table S1. Characteristics of Past Studies in Adults with Moderately-to-Severely Active UC Included in Historical Placebo Control Meta-Analysis**

| **Therapy**  **(Study Name)** | **Population** | **Route of Administration/Induction Dosing Strategy** | **Timing of Clinical Remission Endpoint** | **Clinical Remission Definition** | **Placebo Point Estimate**  **N=988** |
| --- | --- | --- | --- | --- | --- |
| Golimumab (PURSUIT-IV) | Naïve to TNF | IV Week 0 | Week 6 | Mayo score ≤2 with no individual subscore >1 | 11.0% (N=73) |
| Golimumab (PURSUIT-SC) | Naïve to TNF | SC Weeks 0, 2 | Week 6 | Mayo score ≤2 with no individual subscore >1 | 7.2% (N=320) |
| Infliximab (ACT 1) | Naïve to TNF | IV Weeks 0, 2, 6 | Week 8 | Mayo score ≤2 with no individual subscore >1 | 14.9% (N=121) |
| Infliximab (ACT 2) | Naïve to TNF | IV Weeks 0, 2, 6 | Week 8 | Mayo score ≤2 with no individual subscore >1 | 5.7% (N=123) |
| Adalimumab (ULTRA 1) | Naïve to TNF | SC 160 mg at Week 0, 80 mg at Week 2, then 40 mg QOW starting at Week 4 | Week 8 | Mayo score ≤2 with no individual subscore >1 | 9.2% (N=130) |
| Adalimumab (ULTRA 2) | Naïve to TNF subset | SC 160 mg at Week 0, 80 mg at Week 2, then 40 mg QOW starting at Week 4 | Week 8 | Mayo score ≤2 with no individual subscore >1 | 11.0% (N=145) |
| Vedolizumab (GEMINI 1) | Naïve to TNF subset | IV Weeks 0, 2 | Week 6 | Mayo score ≤2 with no individual subscore >1 | 6.6% (N=76) |

Abbreviations: IV, intravenous; QOW, every other week; SC, subcutaneous; TNF, tumor necrosis factor; UC, ulcerative colitis.

**Supplemental Methods. Historical Placebo Control Meta-Analysis**

The meta-analysis for the historical placebo control used the “metafor” package in R^7^. The meta-analytic models were fitted with the rma.uni() function.

For a set of i = 1, 2, …k independent studies, the fixed-effects model (method=“FE”) with no covariates (mods=~1) was fitted to the data using the rma.uni() function with:

rma(yi=p,sei=se,mods=~1,method=“FE”)

Weighted estimation (with inverse-variance weights) is the default and was used where
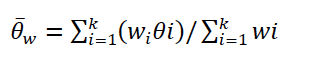


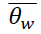
is the weighted average of the true outcomes in the set of k studies, with weights equal to w_i_=1/v_i_ (the “inverse-variance” method).

The placebo point estimate for clinical remission based on the meta-analysis using fixed-effects model with inverse-variance weighting was 8.3% (95% confidence interval [CI], 6.6% to 10.0%).

A sample size of 60 patients in the golimumab arm would ensure that the lower bound of the 90% CI for the golimumab remission rate in children is above 10.0% (i.e., the upper bound of the 95% CI for the historical placebo control), as long as the observed remission rate is ≥18.3%. The probability of observing a remission rate of ≥18.3%, given different assumptions for the true rate of clinical remission (by the Mayo score) is as follows:

- 50% if clinical remission rate of 17.8% (remission rate observed in golimumab-treated adults with ulcerative colitis (UC) in the PURSUIT-SC study) is assumed
- 68% if clinical remission rate of 20.0% is assumed
- 82% if clinical remission rate of 22.5% is assumed
- 91% if clinical remission rate of 25.0% is assumed
- >99.9% if clinical remission rate of 42.9% (remission rate observed in golimumab-treated children with UC in the PURSUIT PEDS PK study) is assumed

Thus, with 60 golimumab-treated patients, the probability of observing a remission rate of ≥18.3% ranged from 50% to greater than 99%. It is reasonable to assume that the true remission rate in children with UC is greater than the remission rate observed in the adult UC study, as the remission rate in the first golimumab UC study (PURSUIT PEDS PK) in children was at least twice that observed in the adult UC study. If we assume the true remission rate to be 22.5%, then the probability of observing a remission rate of ≥18.3% would be greater than 80%.

| **Treatment-emergent AE** | **Week 0 to Week 54**  **N=69** |
| --- | --- |
| UC (worsening/exacerbation) | 42 (60.9)^a^ |
| COVID-19 | 13 (18.8) |
| Upper respiratory tract infection | 12 (17.4) |
| Headache | 12 (17.4) |
| Anemia | 9 (13.0) |
| Abdominal pain | 8 (11.6) |
| Hematochezia | 7 (10.1) |
| Pyrexia | 7 (10.1) |
| Acne | 6 (8.7) |
| Diarrhea | 5 (7.2) |
| Influenza | 5 (7.2) |
| Respiratory tract infection | 5 (7.2) |
| Arthralgia | 5 (7.2) |
| Nasopharyngitis | 5 (7.2) |
| Nausea | 4 (5.8) |
| Vomiting | 4 (5.8) |
| Fatigue | 4 (5.8) |
| Cough | 4 (5.8) |

**Table S2. Common Treatment-emergent AEs (Frequency ≥5% from Week 0 to Week 54)**

Values are n (%), where n is the number of patients with ≥1 treatment-emergent event; patients were counted only once for any given event, regardless of the number of times they actually experienced the event. AEs were coded using MedDRA Version 26.1.

^a^One event of UC was considered “reasonably related” to study drug as assessed by the investigator.

Abbreviations: AE, adverse event; MedDRA, Medical Dictionary for Regulatory Activities; UC, ulcerative colitis.

**Table S3. Key Clinical and Endoscopic Outcomes at Week 6 by Serum Golimumab Concentration Quartiles at Week 6**

| **Outcome at Week 6^a^** | **Serum Golimumab Concentrations at Week 6** | | | |
| --- | --- | --- | --- | --- |
|  | **<1^st^ Quartile^b^**  **n=15** | **≥1^st^ and <2^nd^ Quartile^b^**  **n=15** | **≥2^nd^ and <3^rd^ Quartile^b^**  **n=15** | **≥3^rd^ Quartile^b^**  **n=16** |
| Clinical remission (Mayo)^c^ | 13.3 | 33.3 | 20.0 | 62.5 |
| Clinical remission (PUCAI)^d^ | 20.0 | 33.3 | 20.0 | 56.3 |
| Clinical response^e^ | 26.7 | 60.0 | 46.7 | 87.5 |
| Endoscopic improvement^f^ | 33.3 | 33.3 | 26.7 | 68.8 |

Values are %.

^a^Patients who had a prohibited change in UC medication, an ostomy or colectomy, or discontinued study agent due to lack of efficacy or an AE of worsening of UC before the Week 6 visit were considered not to have achieved the endpoint; data after a discontinuation of study agent due to COVID-19-related reasons (excluding COVID-19 infection) were used as available.

^b^1^st^ quartile=0.93 μg/mL, 2^nd^ quartile=1.56 μg/mL, 3^rd^ quartile=2.78 μg/mL.

^c^Clinical remission (Mayo) was defined as a Mayo score ≤2 points, with no individual subscore >1; patients who had all 4 Mayo subscores missing at Week 6 were considered not to have achieved the endpoint.
^d^Clinical remission (PUCAI) was defined as a PUCAI score <10; patients who had >3 PUCAI subscores missing at Week 6 were considered not to have achieved the endpoint.

^e^Clinical response was defined as a Mayo score decrease from baseline of ≥30% and ≥3 points, with either a decrease from baseline in the rectal bleeding subscore of ≥1 or a rectal bleeding subscore of 0 or 1, based on Mayo endoscopy subscore assigned by the local endoscopist; patients who had all 4 Mayo subscores missing at Week 6 were considered not have achieved the endpoint.
^f^Endoscopic improvement was defined as a Mayo endoscopy subscore of 0 or 1, based on Mayo endoscopy subscore assigned by the local endoscopist; patients who had a missing endoscopy score at Week 6 were considered not to have achieved the endpoint.
Abbreviations: AE, adverse event; PUCAI, Pediatric Ulcerative Colitis Activity Index; UC, ulcerative colitis
